# Modulation of Intracellular Calcium Levels by Calcium Lactate Affects Colon Cancer Cell Motility through Calcium-Dependent Calpain

**DOI:** 10.1371/journal.pone.0116984

**Published:** 2015-01-28

**Authors:** Pasupathi Sundaramoorthy, Jae Jun Sim, Yeong-Su Jang, Siddhartha Kumar Mishra, Keun-Yeong Jeong, Poonam Mander, Oh Byung Chul, Won-Sik Shim, Seung Hyun Oh, Ky-Youb Nam, Hwan Mook Kim

**Affiliations:** 1 Gachon Institute of Pharmaceutical Sciences, Gachon University, Incheon 406–840, Republic of Korea; 2 Lee Gil Ya Cancer and Diabetes Institute, Department of Molecular Medicine, Gachon University, Inchon 406–840, Republic of Korea; 3 Department of Zoology, School of Biological Sciences, Dr. Harisingh Gour Central University, Sagar 470003, India; University of Birmingham, UNITED KINGDOM

## Abstract

Cancer cell motility is a key phenomenon regulating invasion and metastasis. Focal adhesion kinase (FAK) plays a major role in cellular adhesion and metastasis of various cancers. The relationship between dietary supplementation of calcium and colon cancer has been extensively investigated. However, the effect of calcium (Ca^2+^) supplementation on calpain-FAK-motility is not clearly understood. We sought to identify the mechanism of FAK cleavage through Ca^2+^ bound lactate (CaLa), its downstream signaling and role in the motility of human colon cancer cells. We found that treating HCT116 and HT-29 cells with CaLa immediately increased the intracellular Ca^2+^ (iCa^2+^) levels for a prolonged period of time. Ca^2+^ influx induced cleavage of FAK into an N-terminal FAK (FERM domain) in a dose-dependent manner. Phosphorylated FAK (p-FAK) was also cleaved in to its p-N-terminal FAK. CaLa increased colon cancer cells motility. Calpeptin, a calpain inhibitor, reversed the effects of CaLa on FAK and pFAK cleavage in both cancer cell lines. The cleaved FAK translocates into the nucleus and modulates p53 stability through MDM2-associated ubiquitination. CaLa-induced Ca^2+^ influx increased the motility of colon cancer cells was mediated by calpain activity through FAK and pFAK protein destabilization. In conclusion, these results suggest that careful consideration may be given in deciding dietary Ca^2+^ supplementation to patient undergoing treatment for metastatic cancer.

## Introduction

Colon cancer is the third leading cause of cancer-related mortality worldwide, accounting for over 600,000 deaths every year with increasing incidence. Carcinogenic induction in the colon occurs through a sequence of events leading to metastasis involving various oncogenic proteins. Focal adhesion kinase (FAK) plays a critical role in colon cancer progression and is an important oncogenic protein involved in cell proliferation, survival and motility [1,2]. Calpain is a well-conserved cysteine protease activated by increased intracellular Ca^2+^ (iCa^2+^). It localizes to focal adhesion, and potentially induces cleavage of focal adhesion proteins. Comparatively, metastatic tumors contain higher levels of calpain than non-metastatic tumors. Calpain-mediated FAK degradation is critical for motility in human colon cancer cells [3]. It appears that the function of calpain in cell adhesion and motility limits their ability to cleave components of focal complexes, which in turn may increase adhesion turnover and functionally significant tumor progression [4,5].

The iCa^2+^ ion is an important signaling molecule that modulates numerous cellular processes in cancer physiology, including cell motility. The iCa^2+^ is maintained at a low concentration (~100 nM) compared to the extracellular free Ca^2+^ (1.2 mM) [6,7]. Low levels of cytoplasmic Ca^2+^ is maintained through active efflux from cells via the plasma membrane Ca^2+^ ATPase pump, whereas sarcoendoplasmic reticular Ca^2+^ ATPase removes Ca^2+^ by proton exchange. Other Ca^2+^ permeable channels such as stored-operated calcium channels, transient receptor potential (TRP) channels and the calcium release activated channel (CRAC) protein 1 are also involved in the iCa^2+^ homeostasis. Remodeling or deregulation of iCa^2+^ homeostasis in cancer cells causes changes in cancer progression [8]. Dietary supplementation of Ca^2+^ was known to reduce the risk of colorectal adenomas and cancer [9]. However, the impact of Ca^2+^ dietary supplementation remains unknown on cancer metastasis.

Normal cells have a low concentration of extracellular lactate ranging between 0.5 and 2 mM, but the concentration for cancer cells can be as high as 30–40 mM [10]. Cancer cell survival requires an alkaline intracellular environment and acidic extracellular environment [11,12]. Monocarboxylate transporters (MCTs) primarily control the movement of intracellular and extracellular lactic acid [13]. Elevated levels of MCT1 have been detected in breast, colon, gastric, and cervical cancers. MCT4 expression is highly elevated in renal carcinoma, cervical and prostate cancers [14]. MCT4 releases lactate in response to hypoxia, whereas lactate uptake into oxygenated tumor cells occurs via MCT1 [15,16]. Lactate acts as a substrate for oxidative metabolism in oxygenated tumor cells.

Based upon the information on Ca^2+^ dietary supplementation in metastatic cancer, we investigated the motility of colon cancer cells by direct exposure of Ca^2+^ bound lactate (CaLa). We focused on the calpain-FAK-cell pathway to understand underlying molecular mechanisms of human colon cancer cell motility.

## Materials and Methods

### Cell cultures

All the cell lines were purchased from American Type Culture Collection (Manassa, Va). Human colon cancer cell lines HCT116, HT-29, and DLD1were cultured in RPMI1640 medium. Normal colon cell line CCD-18Co was cultured in DMEM medium supplemented with 10% fetal bovine serum and 1% penicillin-streptomycin in a humidified atmosphere at 37°C containing 5% CO_2_.

### Reagents and antibodies

CaLa, FAK inhibitor (TAE226), calpain inhibitor (Calpeptin), Flu-3AM was purchased from Sigma (St. Louis, Mo). Primary antibodies used for western blot analysis (FAK, pFAK, calpain-2, p53, MDM2, pMDM2, α-tubulin, Lamin B, GADPH) and the corresponding secondary antibodies were purchased from Cell Signaling Technology (USA).

### Thermodynamics of divalent metal ion binding to lactic acid

To measure the binding isotherms for interaction of the bivalent metal ion Ca^2+^ to lactic acid, isothermal titration calorimetry (ITC) experiments were performed using a Microcal 200 isothermal titration microcalorimeter (Microcal, Inc., Northampton, MA). Data collection, analysis, and plotting were performed using the Windows-based software package Origin (version 7.0; supplied by Microcal). The titrating microcalorimeter contained sample and reference cells held in an adiabatic enclosure. The reference cell was filled with distilled water. Typical titrations involved injecting 1.5 μL of 5 mM of Ca^2+^ ion (using a computer-controlled injector) into the sample cell (filled with 0.5 mM of lactic acid at pH 7.0). Samples were injected at 2-min intervals to ensure that the titration curves had returned to baseline prior to the next injection. The syringe stir rate was set to 1,000 rpm. The absorption or release of heat during each injection was measured using the calorimeter. Titration isotherms for the binding interactions comprised the differential heat flows for the different injections. These values were then integrated to determine the enthalpy change associated with each injection. Heat changes caused by the injection of each metal ion into distilled water were negligible. The dilution data were subtracted from the sample data and bad data points were removed. The data fitting was performed using Origin software (version 7.0) and the fitting process was titrated until the best fit determined by the chi-square minimization method was obtained. Fitting the binding isotherms in this way provided data on the binding constant (Ka), change in enthalpy (∆H), and stoichiometry of binding (n). The binding Gibbs free energy (∆G) was calculated from the enthalpy change (∆H) and binding constant (Ka) using the equation: ∆G = RTlnKa = ∆H T∆S, where R is the gas constant and T is the absolute temperature in degrees Kelvin [17]. Meanwhile, the stoichiometry’s (n) of the different interactions were determined from the One Set of Sites models.

### Measurement of iCa^2+^ concentration

Cytosolic free Ca^2+^ ion concentrations were measured using a Confocal Laser-Scanning Microscope (Leica, Heidelberg, Germany). Cultured HCT116 and HT-29 cells were loaded with 10 μM Fluo-3/AM and 1μl 25% Pluronic F-127 (dissolved in DMSO) and incubated for 30 minutes at 37°C. After loading fluorescence probes, 2.5 mM of CaLa was added and the image was acquired. Fluo-3/AM was excited at 488 nm and emitted fluorescence was measured at 515 nm. Intracellular Ca^2+^ levels are expressed as F/F0 ratios, where F0 is the initial fluorescence intensity (Image J software analysis).

### Wound healing assay

For wound healing assay, ibidi culture insert consisting of two reservoirs separated by a 500 μm thick wall was used. Cells were seeded into two-chamber (100 μl of 4×10^5^ cells/ml) ibidi culture insert. After 24 h incubation, the chamber was gently removed creating a gap of ~500 μm. Cells were then allowed to migrate into the bared areas for 6 h. The live cell imaging was done using the JuLi Br, Live cell analyzer (NanoEnTek Inc, South Korea).

### Western blot analysis

Cellular lysates were prepared and an equal amount of protein was separated by SDS PAGE and blotted on to polyvinylidene fluoride (PVDF) membranes as described earlier [18,19,20]. Specific primary antibodies followed by HRP-conjugated secondary antibody were used for detecting specific proteins using ECL system (AbClon) for signal detection.

### Immunofluorescence Analysis

HCT116 and HT-29 cells were treated with CaLa for 12h. Cells were fixed in 100% cold ethanol for 20–30 min at-20°C, permeabilized with 0.3% triton x100 in PBS for 15 min at room temperature, and washed serially in PBS. Cells were blocked with 3% horse serum in PBS for 1h at room temperature and washed with PBS. Cells were then incubated with FAK antibody (1:800) with 3% horse serum in PBS at 4°C overnight. Cells were washed with PBS and subsequently incubated with Alexa-488 conjugated secondary antibody for 1h at room temperature. Cells were then sealed with mounting media containing DAPI (Vectashield, Vector Laboratories). Images were captured using confocal laser scanning microscopy (CLSM, Nikon A1+, Japan).

### Statistical analyses

All the data were presented as the mean ± standard deviation (SD). Statistical significance was evaluated using Student’s t-test and *P* < 0.05 was considered significant.

## Results

### Lactic acid binds to calcium ions with high affinity

**Figure 1:**
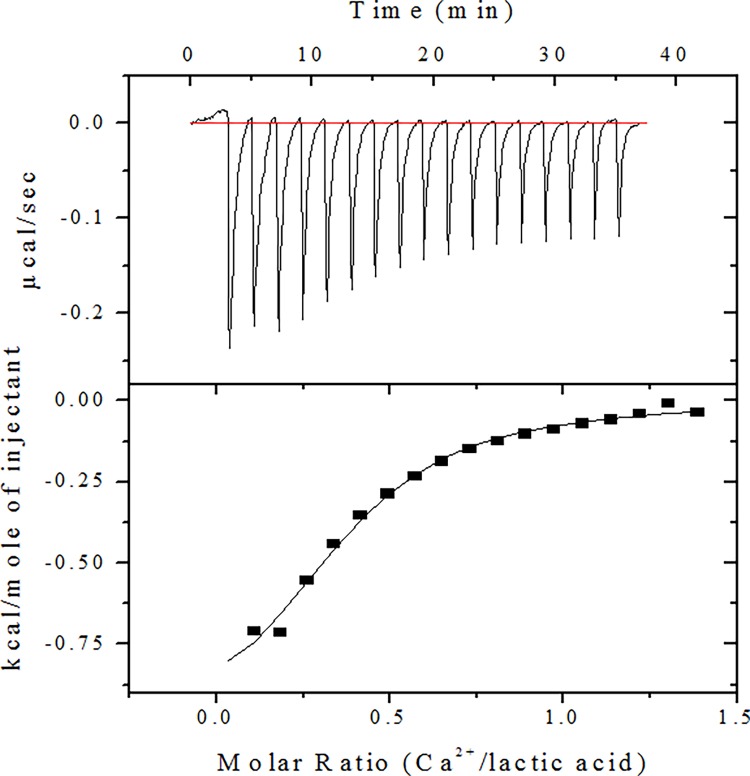
Isothermal titration calorimetric analysis of lactic acid binding with calcium. Three representative isotherms are shown illustrating binding as a function of pH at 37°C. Upper and lower panels show the results of calorimetric titration of 1.5 μL of 5 mM Ca^2+^ and 0.5 mM lactic acid mixture in 5 mM Tris Hcl at pH 7.0. For each titration, raw data are shown in the upper panel and integrated heat data are shown in the lower panel.

We analyzed the thermodynamic properties of CaLa and binding interactions between Ca^2+^ and lactic acid by isothermal titration calorimetry (ITC). Representative ITC data relating to the binding of lactic acid to Ca^2+^ at pH 7.0 is shown in Fig. 1. Further details including the calculated association constants and enthalpy and entropy of associations are listed in Table 1. Results show that lactic acid binds to Ca^2+^ with K_d_ 6.2×10^−5^ M, a thermodynamically suitable condition to form CaLa salt. Our thermodynamic calculations showed that one lactate molecule binds to 0.361 Ca^2+^ ions, even though two-lactate molecule binds to one Ca^2+^ ions in theory. The ITC data demonstrates the possibility that extracellular lactic acid in the form of lactate can form CaLa salt, which may exist as a soluble form under the aqueous solubility limit concentration (7.9g /100 ml). This data also suggest that CaLa can be used to administer intracellular calcium, which may dissociate under variable milieu.

**Table 1:** Thermodynamic parameters of lactic acid binding to Ca^2+^ at pH 7.0 determined by ITC^a^.

Parameters	Ca^2+^
K_d_	6.2 ×10^−5^
n	0.361
∆G (Kcal/mol-1)	−5.97
∆H (cal/mol-1)	−1,098
∆S (cal/mol/deg-1)	15.7

^a^ The experiments were performed at 310 K with a 0.5 mM lactic acid solution at pH 7.0. The n value represents the number of bound metal ions per lactic acid molecule. The thermodynamic parameters are given for the association reaction.

### CaLa administration increased iCa^2+^ levels

**Figure 2:**
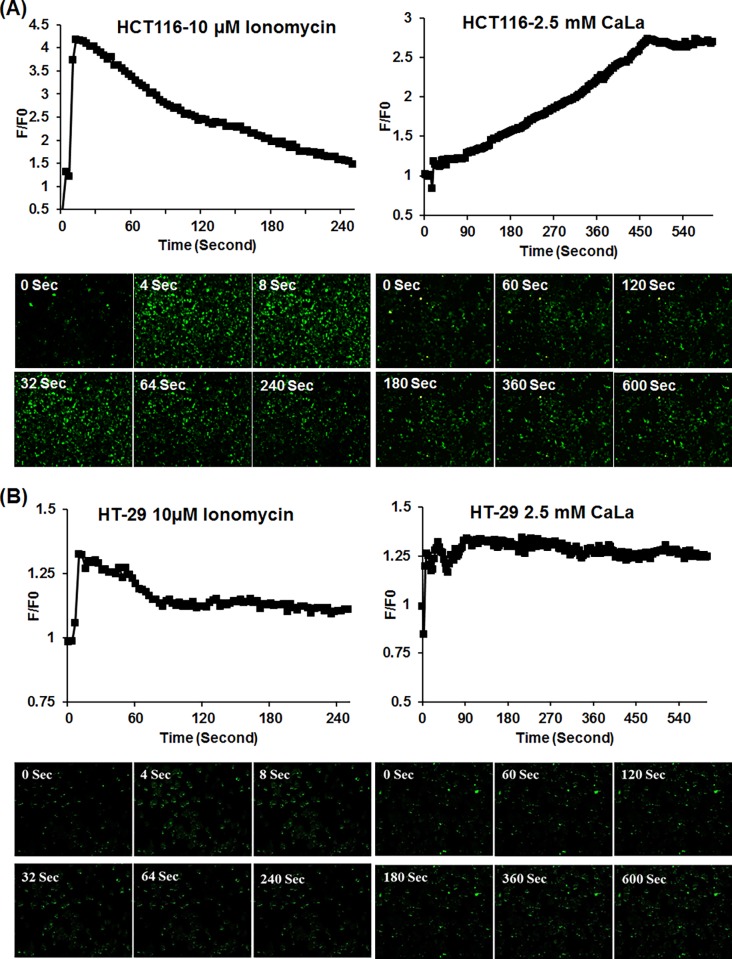
Determination of intracellular Ca^2+^ levels in colon cancer cells. Cultured HCT116 (A) and HT-29 (B) cells were treated with Fluo-3AM fluorescence probes as described in material and methods. Cells were subsequently incubated with 2.5 mM CaLa and 10 μM Ionomycin in DMSO (control). Image was acquired at a time point of 0 seconds as initial and 600 seconds as maximum using Confocal Laser-Scanning Microscope (Leica, Heidelberg, Germany). The Ca^2+^ fluorescence image was converted into fluorescence intensity and plotted as the indicated F/F0 (Image J software analysis). F0 is the initial fluorescence intensity.

We assessed the effect of CaLa administration on colon cancer cells and analyzed the levels of dissociated iCa^2+^. HCT116 and HT-29 cells were treated with 2.5 mM CaLa that showed a remarkable difference at 0 and 600 seconds in both cell lines. The experimental validation was done by using ionomycin, an ionophore used to raise iCa^2+^ levels. Ionomycin (10 μM) drastically increased iCa^2+^in the first few seconds, followed by a gradual decreased in both cell lines (Fig. 2). Although ionomycin raised iCa^2+^ levels, these results suggest that ionomycin is not stable in colon cancer cell lines. However, in HCT116 cells, CaLa gradually increased iCa^2+^ to its maximum level at 480 seconds and then plateaued (Fig. 2A). HT-29 cells reached to maximum iCa^2+^ concentration between 50 and 100 seconds, and then plateaued (Fig. 2B). These results suggested that CaLa increased iCa^2+^ levels and maintained those levels for a prolonged period of time.

### CaLa modulated FAK stability

**Figure 3:**
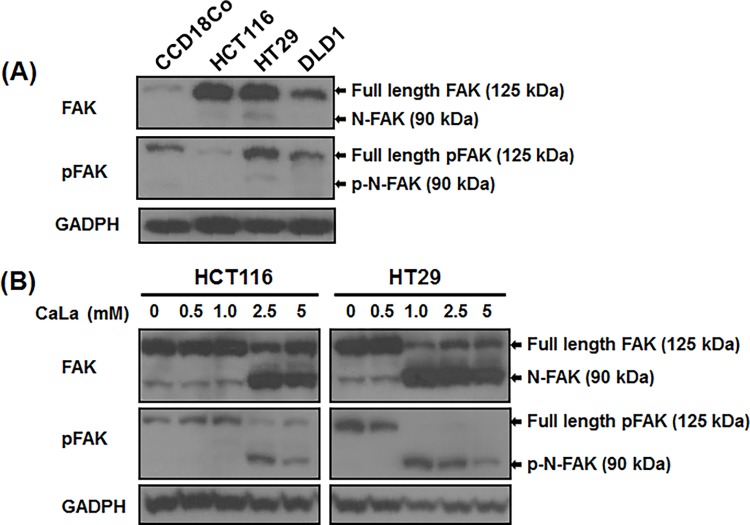
CaLa induced FAK and pFAK cleavage in colon cancer cells. (A) FAK expressions were analyzed in the normal colon epithelial cell line CCD-18Co and different colon cancer cell lines (HCT116, HT-29 and DLD1). HCT116 and HT-29 (B) were treated with different concentrations of CaLa for 12 h. Western blotting performed from cell lysates showed that CaLa induced FAK and pFAK cleavage in a dose-dependent manner.

Recent studies indicate that overexpression of the oncogene FAK is associated with carcinogenesis of various human cancer cells [21,22]. The iCa^2+^ activates calpain, which degrades FAK and enhances the motility of cancer cells [3,4,5,9]. Having observed that CaLa increases iCa^2+^ levels in colon cancer cell lines, we investigated the effect of CaLa on FAK stability in these cells. We examined the expression of FAK and pFAK in normal colon (CCD-18Co) and colon cancer cell lines (Fig. 3A). Results showed that CCD-18Co cells have nominal expression of FAK and thus lower levels of pFAK as compared to colon cancer cell lines expressing high levels of FAK and pFAK. Each colon cancer cell line showed different expression levels of FAK and pFAK. CCD-18Co cells showed a normal level of pFAK. HT-29 and DLD-1 cells showed higher levels of pFAK as compared to HCT116. Therefore, we focused on HCT116 and HT-29 cell lines for further studies related to FAK protein stability. In addition, we performed a dose-dependent analysis for the effects of CaLa on HCT116 and HT-29 cells (Fig. 3B). Cells were treated with different concentration of CaLa for 12 h and FAK cleavage was assessed. The full length of FAK and pFAK is a 125 kDa. CaLa cleaved FAK into a 90 kDa protein N-FAK (FERM domain) with increasing concentrations having the highest activity at 2.5 mM. Similarly, pFAK was cleaved in to its p-N-FAK in a dose-dependent manner with optimal activity at 2.5 mM of CaLa (Fig. 3B). These results indicate that CaLa destabilizes FAK and pFAK and causes cleavage of both proteins in colon cancer cells.

### FAK destabilization through caplain activity by CaLa

**Figure 4:**
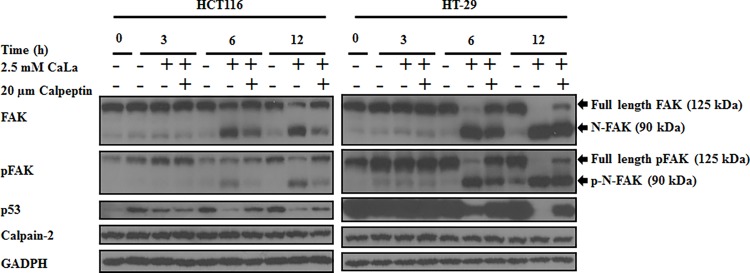
CaLa causes calpain-mediated FAK cleavage in human colon cancer cells. HCT116 and HT-29 cells were treated with 2.5 mM CaLa in combination with the calpain inhibitor (calpetin 20 μM) in the indicated time-dependent manner. Western blot data demonstrate that calpain inhibitor, calpeptin, reduced the FAK and pFAK cleavage as well as p53 degradation.

Recent studies indicate that FAK and its phosphorylated form are important for focal adhesion turnover. FAK is cleaved by calpain, a Ca^2+^ dependent protease, which plays a critical role in focal adhesion dynamics in motile cells [4]. Calpain is involved in several key aspects of cancer cell adhesion, migration, and metastasis [23]. Since CaLa increased Ca^2+^ influx and cleaved FAK, so we determined the effects of CaLa on calpain in HCT116 and HT-29 cells (Fig. 4). Results show that CaLa caused time-dependent cleavage of FAK and pFAK proteins but there was no effect on the levels on calpain-2 in both cell lines. To confirm the results, we used calpeptin, a rho kinase activator and an inhibitor of calpain that is part of a family of calcium-dependent cysteine proteases. Calpeptin (20 μM) reversed the effects of CaLa on FAK and pFAK cleavage in both cell lines with no effects on calpain-2 protein levels (Fig. 4). These data suggest that CaLa increased Ca^2+^ influx and calpain activity but not its protein levels. It is known that cleaved FAK mediates degradation of p53 through nuclear translocation of N-FAK. Cells treated with CaLa showed decreased levels of p53, which was reversed by calpeptin. Calpeptin inhibited calpain-2, preventing it from cleaving FAK, attenuating p53 degradation. These results established that CaLa-induced FAK cleavage enhanced p53 degradation and calpain-2 activity but without effects on calpain protein levels.

### Cleaved FAK translocates into nucleus

**Figure 5:**
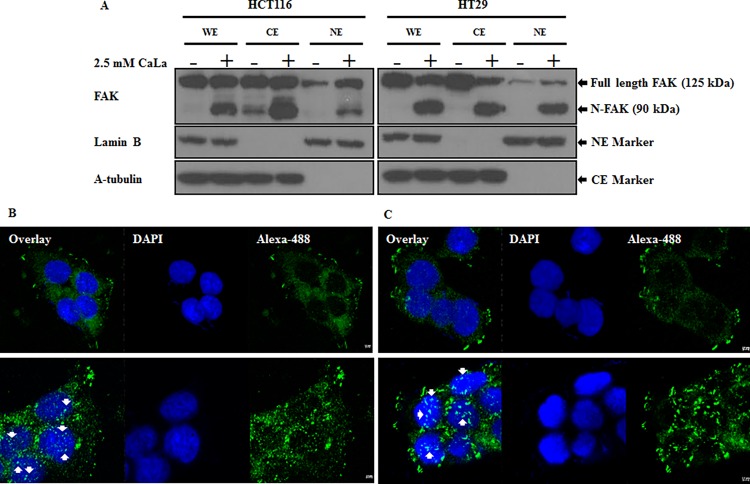
FAK is cleaved by CaLa and translocates into the nucleus. Translocation of N-FAK was analyzed by western blot (A) and immunocytochemistry (B-C). HCT116 and HT-29 cells were seed in a 8-well chamber and treated with 2.5 mM CaLa for 12 h. Translocation was analyzed by confocal microscopy. WE, whole cell extraction; CE, cytoplasmic extraction; NE, nuclear extraction; translocated N-FAK is indicated by an arrow. Scale bar: 2.5 μM.

Studies showed that translocation of nuclear FAK promoted cell survival through enhanced p53 degradation under conditions of cellular stress [24,25]. CaLa induced cleavage of FAK into a 90-kDa protein, which was translocated into the nucleus. Fig. 5 represents the western blot analysis from whole, cytoplasmic, and nuclear fractions of cells treated with CaLa, and their immunocytochemistry. The whole cell lysate and cytoplasmic fraction of both cell lines treated with CaLa showed FAK and cleaved FAK. Nuclear fractions of untreated cells had low levels of FAK but treatment with CaLa increased FAK and cleaved FAK levels in nuclear fractions (Fig. 5A). The nuclear translocations of N-FAK in HCT116 and HT-29 (Fig. 5 B and C) were visualized after incubation with anti-FAK primary antibody followed by staining with Alexa-488 conjugated secondary antibody. The confocal microscopy of control cells showed DAPI stained nuclei without Alexa-488-stained FAK. However, confocal imaging of CaLa-treated cells showed translocation of Alexa-488 stained N-FAK into DAPI stained nuclei. These results clearly indicate that 2.5 mM CaLa cleaved full length FAK that translocated into the nucleus.

### CaLa increased the motility of colon cancer cells

**Figure 6:**
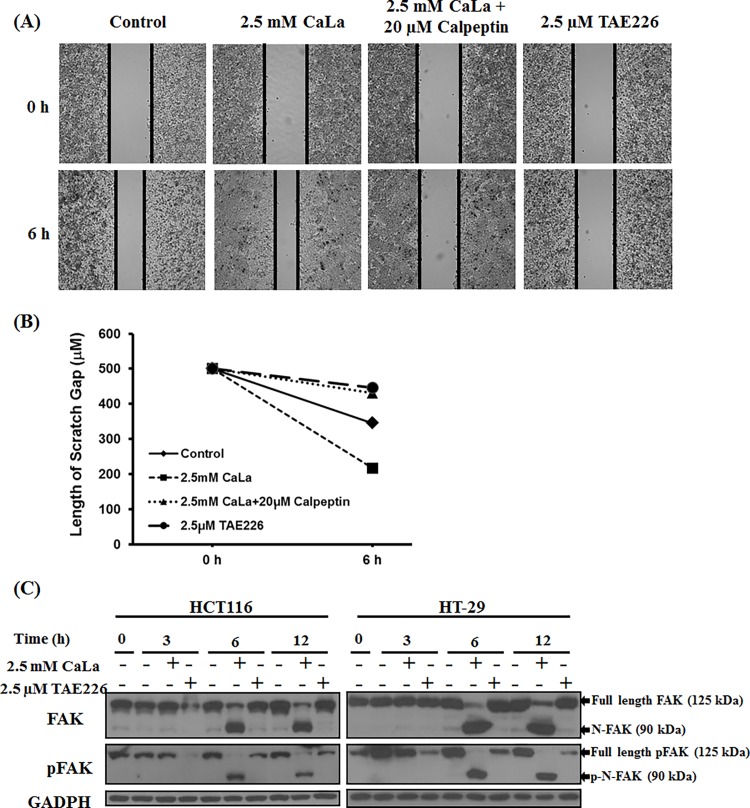
CaLa increased colon cancer cell motility. Wound healing assay was performed in HCT116 cells. Cells were treated with CaLa, and wound healing property was analyzed from 0 to 6 h after wound (A), and mean values of wound area were analyzed using multiple line graph (B). HCT116 and HT-29 cells were treated with 2.5 mM of CaLa in the time-dependent manner induced FAK and pFAK cleavage (C). pFAK inhibitor (TAE226 2.5 μM) reduced pFAK levels as detected by western blot.

Since FAK is known to play a critical role in adhesion, migration, proliferation and differentiation in cancer cells [1,2], we studied the effects of CaLa on these cellular properties in colon cancer cells. We performed wound healing assay in HCT116 cells treated with CaLa alone or in combination with calpeptin (Fig. 6). Results demonstrate that CaLa (2.5 mM) increased the motility of HCT116 cells. Calpeptin alone did not appear to change the motility. Interestingly, it was shown that application of calpeptin (20 μM) in combination with CaLa reduced the effect of CaLa and inhibited cell migration (Fig. 6 A–B). Further, we observed that TAE226 (FAK inhibitor) significantly decreased the cell motility. These results suggest that Ca^2+^ influx mediates FAK degradation through calpain activity. TAE226 showed anti-tumor effects in colon cancer cells. It reduced the size of disseminated tumors and prolonged survival in tumor-bearing mice [26]. In order to confirm our observations, we performed a comparative analysis of FAK and pFAK levels in HCT116 and HT-29 using TAE226 (Fig. 6C). TAE226 (2.5 μM) inhibited pFAK expression in a time-dependent manner did not inhibit total FAK. CaLa-induced FAK and pFAK cleavage increased motility compared to TAE226, which suppressed pFAK activity and decreased cancer cell motility.

### Cleaved-FAK mediated p53 degradation through MDM2-dependent ubiquitination

**Figure 7:**
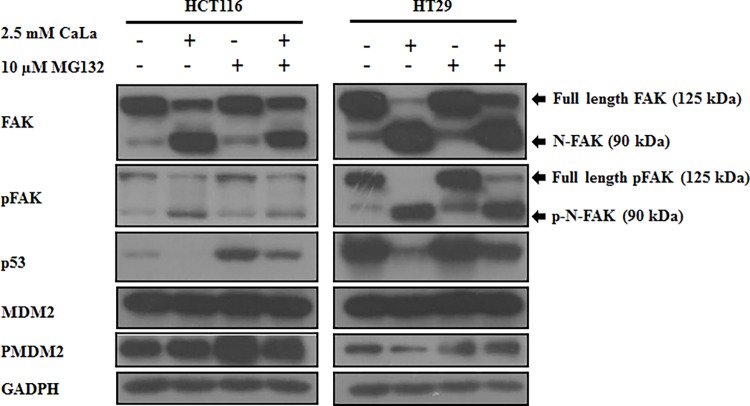
Cleaved FAK inhibits p53 activity through Mdm2-dependent ubiquitination and proteasomal degradation. HCT116 and HT-29 cells were treated with 2.5 mM CaLa in combination with a proteasomal inhibitor (MG132 10 μM) for 10 h. Western blot was performed from cell lysates and proteins were detected as described in methods.

Previous studies indicated that the structure of FERM-FAK can translocate into nucleus and modulate p53 protein stability through MDM2 [25]. Therefore, we investigated the mechanism of p53 down regulation by CaLa. CaLa alone induced cleavage of FAK and pFAK. Treatment of MG132 with CaLa paradoxically recovered the FAK and pFAK cleavage (Fig. 7). Treatment of cells with CaLa inhibited p53 levels while MG132 recovered the levels of p53. Treatment of MG132 in combination with CaLa recovered the effect of CaLa on p53 proteosomal degradation. In addition, we observed that MDM2 and PMDM2 protein levels were not changed by treatments with CaLa, MG132 or both. Thus, these results suggest that MG132 recovered p53 from CaLa through MDM2-associated ubiquitination.

## Discussion

This study demonstrates that calcium lactate-induced Ca^2+^ influx increases the motility of colon cancer cells through destabilization of FAK and pFAK proteins. This study demonstrates the effect of iCa^2+^ on the calpain-FAK-cell motility pathway. CaLa-mediated cleavage of FAK and pFAK was arbitrated through calpain activity, which increased the motility of colon cancer cells. The cleaved FAK translocated into the nucleus and enhanced p53 degradation as well as calpain activity. The effect of extracellular lactate was reported in several studies about the motility of cancer cells. One major difference between the present study and previous studies on lactate is the chemical entity.

Monocarboxylate transporters (MCTs) usually mediate lactate influx and efflux and that elevated levels of MCT1 have been detected in many cancers, including colon cancer [16]. Lactate release in hypoxic cells is mediated through MCT4, whereas the lactate uptake into oxygenated tumor cells is mediated via MCT1 [15,16]. It appears that CaLa itself is being transported into the cytoplasm, thus, increasing iCa^2+^ and lactate concentrations are coincidently observed. However, the specific transport mechanism is not identified. The present results showed that low concentration of CaLa mediated the FAK-dependent motility in colon cancer cells. Previous studies indicated that sodium lactate was used in concentrations as high as 20 mM [10]. This high range was observed in malignant and metastatic tumors of large sizes. High concentrations of lactate in tumors were correlated with a high incidence of distant metastasis and other malignant behaviors of cancer cells [27]. Therefore, we used low concentrations of lactate to avoid cancer metastasis. By treating cells with low concentrations of CaLa, Ca^2+^ influx increased and maintained for a prolonged period of time, which increased calpain activity. The intracellular thiol protease calpain catalyzes the limited proteolysis of various focal adhesion structural proteins and signaling enzymes in adherent cells. Calpains are involved in several key aspects of migration, including adhesion and spreading. Pharmacological inhibition of calpains reduced integrin-mediated cell migration [23]. Recent studies provide further support for calpain as an important modulator of cell motility through its capacity to regulate focal adhesion dynamics. Calpain-mediated FAK degradation is critical for motility in human colon cancer cells [3]. High levels of FAK expression have been associated with increased invasiveness in malignant tumors. However, the mechanisms of FAK activation, cleavage, and deregulation of motility, are not clear. Signaling events downstream of FAK activation and cleavage may contribute to the motility of human colon carcinoma cells.

CaLa can modulate the migratory potential of oxygenated tumor cells, which exist around tumor vessels. In tumor tissue, the vascularity was limited to supply oxygen and nutrients such as glucose and glutamine. Hypoxic tumors appear to be poorly differentiated but hypoxia plays a direct role in the maintenance of cancer stem cells [29]. Hypoxic tumors tend to produce a lactate gradient, which is used by oxygenated tumor cells through MCT1 mediated transportation, so the hypoxic tumor cells can utilize glucose. This phenomenon is known as the intercellular lactate shuttle. Accordingly, it could be assumed that in the extracellular environment surrounding hypoxic cells gradually accumulates lactate in its free form. However, oxygenated cells were supplied by calcium through the blood supply, lactate was imported via MCT1, lowering the extracellular lactate concentration and its microenvironment contains less lactate and mostly bound to calcium [31]. Our results imply that oxygenated cells are less motile compared with hypoxic cells and that metastasis involves hypoxic cells rather than oxygenated cells. The existence and amount of CaLa in an extracellular tumor microenvironment could be a determining factor for metastasis through modulation of FAK. In addition, CaLa may possibly control cell motility and energy metabolism. The tumor suppressor protein p53 prevents cancer progression through numerous mechanisms including apoptosis and cell cycle arrest. A well-characterized link exists between metabolism and genetic regulation of p53, where p53 regulates the cell’s energy balance between glycolysis and oxidative phosphorylation [30,31]. Similarly, our results show that CaLa can cause proteosomal degradation of p53 (Fig. 4) through nuclear translocation of FERM-FAK and mdm2-mediated ubiquitination (Fig. 7).

The current study investigated the impact of supplemental Ca^2+^ on the molecular mechanisms of metastatic colon cancer. The importance of CaLa in modulation of colon cancer cell physiology is not limited to basic studies but also have an impact on clinical nutritional outcome. Ca^2+^ plays various roles in cancer progression, including increasing apoptosis or inhibiting proliferation in the colon. Furthermore, Ca^2+^ binding to bile salts, increased in high fat diets, have been associated with colonic carcinogenesis [28]. CaLa-induced FAK cleavage and enhanced cell motility of HCT116 cells creates an assumption that metastatic colon cancer patients need more careful when considering Ca^2+^ dietary supplementation. Because extracellular calcium can be ionized with lactate, it leads to Ca^2+^ influx causing increased FAK cleavage and cancer cell motility.

## Conclusions

The present study elucidates that CaLa increased motility of colon cancer cells by calpain activity through destabilizations of FAK and pFAK proteins. The concentration and chemical entity of CaLa may be contributing factor for colon cancer cell motility. Therefore, our results suggest that motility effects on colon cancer may be related to the use of Ca^2+^ dietary supplementation. Even a low concentration of Ca^2+^ may cause increased cell motility.
